# Improving Blood Transfusion Practices in a Community Hospital Setting: Our Experience with Real-Time Clinical Decision Support

**DOI:** 10.3390/medsci6030067

**Published:** 2018-08-22

**Authors:** Muhammad Sardar, Muhammad Azharuddin, Ananta Subedi, Prateek Ghatage, Doantarang Du, Arpad Szallasi

**Affiliations:** 1 Department of Internal Medicine, Monmouth Medical Center, 300 2nd Ave, Long Branch, NJ 07740, USA; Muhammad.sardar@RWJBH.org (M.S.); azhar_uddin203@yahoo.com (M.A.); ananta2226@gmail.com (A.S.); prateek.ghatage@gmail.com (P.G.); Doantarang.Du@RWJBH.org (D.D.); 2 Department of Pathology, Monmouth Medical Center, 300 2nd Ave, Long Branch, NJ 07740, USA

**Keywords:** blood utilization, real-time transfusion support, inappropriate transfusion alert

## Abstract

There is good evidence that 50% or more of red blood cell (RBC) transfusions are unnecessary. To curtail inappropriate RBC transfusions at our hospital, real-time clinical decision support was implemented in our electronic medical record (EMR) that alerts clinicians to the patient’s most recent pretransfusion hemoglobin value upon order entry and provides Best Practice Advisory. This is a soft pop-up alert which is activated when the hemoglobin exceeds 7 g/dL. The ordering clinician can either honor (by cancelling the order) or override the alert. We studied the impact of the alert on blood utilization during a 3-month period (November 2016 to January 2017). For patients who were transfused despite the alert, a retrospective review of the medical chart was performed to determine whether or not the transfusion was clinically indicated. During the study period, 178 of the 895 RBC transfusion orders (20%) triggered the alert. After excluding duplicates, 144 orders were included in our analysis. Most of these orders (124/144, 86%) were carried out despite the alert. According to our chart review, 48% of the alert transfusions could be considered inappropriate, with hemodynamically stable, asymptomatic anemia being the leading indication. Of clinical services, orthopedic surgery had the highest rate of overriding the alert with no clinical justification (70%). The number of RBC transfusions dropped from 313.5 units per month (preintervention period) to 293.2 units per month (postintervention period)—a 6.5% decrease. Real-time clinical decision support may reduce the number of inappropriate RBC transfusions in a community hospital setting, though in our study, the decrease in blood utilization (6.5%) did not reach statistical significance.

## 1. Introduction

A decade ago the Institute of Medicine estimated that a third of all health care spending in the U.S. was wasteful, with blood transfusions being among the leading unnecessary interventions [1]. Indeed, there is good evidence that 50% or more of red blood cell (RBC) transfusions are unnecessary [2]. This estimate is consistent with the much lower transfusion rate in other developed countries [3].

Since transfusions carry inherent risks, reducing the number of inappropriate transfusions has been a major goal of hospital Blood Utilization Committees [4]. In addition, The Joint Commission included patient blood management among its seven major initiatives to improve patient outcomes and the quality of care [5].

Traditional approaches to improve blood utilization rely on retrospective review of blood component use (audits) and resultant education efforts. Although successful initially, this approach, as Goodnough and Shah have recently pointed out, is “enormously labor intensive, is difficult to maintain long term, and can delay product delivery during ongoing conversation between transfusion and clinical services” [6]. There may be a clinical use for a computer-based, real-time approach to guide transfusion decisions in order to improve patient outcomes.

The Affordable Care Act (better known as “ObamaCare”) encouraged the adoption of electronic medical records (EMR) to prevent or at least minimize unnecessary medical treatment. Importantly, EMR includes a new health information technology, referred to as clinical decision support (CDS), which can provide real-time assistance in clinical decision-making. Over the years, CDS has been successfully used to guide diagnostic and therapeutic interventions, including transfusions, in various inpatient and outpatient settings. The Stanford Hospitals and Clinics has built a CDS-based transfusion appropriateness system that triggers an alert when the patient’s pre-transfusion hemoglobin level exceeds a preset value—7 g/dL for most patients and 8 g/dL for those with acute coronary syndrome or post cardiothoracic procedure [6,7]. This system was implemented in 2010 and has resulted in a 24% decrease in the number of annual RBC transfusions, translating into a cumulative net saving of $6.4 million over a 4-year period [6,7]. Capitalizing on the published experience of The Stanford Hospitals and Clinics, in November 2016 we implemented a similar real-time CDS in the EMR of Monmouth Medical Center, Long Branch, New Jersey. Our hospital is a community-based teaching hospital with residency programs in various disciplines, including medicine, surgery, and obstetrics and gynecology (ObGyn). Here we report our experience with the real-time transfusion decision support system.

## 2. Materials and Methods

**Figure 1:**
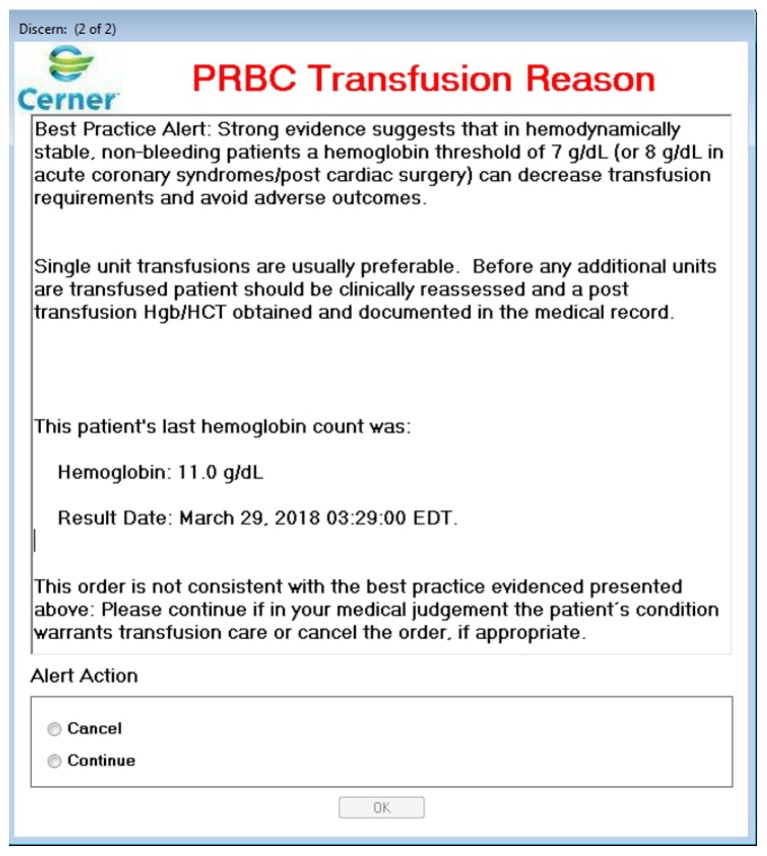
Best Practice Alert to guide red blood cell (RBC) transfusion decisions real-time when the order is placed. The alert appears when the patient’s pre-transfusion hemoglobin value exceeds 7 g/dL. This is a soft alert that the clinician can override by selecting a clinical justification for the transfusion from a drop-box (not shown). The electronic medical record collects data and provides the Blood Bank Director with a bi-weekly summary of the orders that triggered the alert along with the clinicians’ responses.

On 1 November 2016, after educating the medical staff (lecture to residents, email blast to attendings, etc.), real-time CDS was incorporated into the EMR of our institution. When an order for RBC transfusion is placed, the Laboratory Information System (LIS) pulls the patient’s most recent pre-transfusion hemoglobin value. The pop-up alert is triggered when the hemoglobin exceeds 7 g/dL. This is a soft alert: the provider may honor the alert by cancelling the order or can go ahead with the RBC transfusion after selecting an explanation for overriding the alert from a drop-box (Figure 1).

During the 3-month post-intervention study period (1 November 2016 to 31 January 2017), 178 orders activated the alert. After excluding duplicate orders for the same unit of blood, 144 orders were included into our retrospective chart analysis. The chart analysis was performed by four internal medicine residents with the supervision of an attending clinician. The analysis encompassed various factors such as clinical indication for the RBC transfusion, pretransfusion laboratory values (including hemoglobin), and vitals (pulse, blood pressure, etc.). The appropriateness of the transfusion was determined based on the American Association of Blood Banks (AABB) 2016 guidelines [8]. We also analyzed the data according to clinical service (e.g., medicine, surgery) and the level of the provider (nurse practitioner, resident and attending physician).

The preintervention monthly blood utilization was calculated based on the January to October 2016 transfusion records. The postintervention value was determined by the November 2016 to January 2017 transfusion records. Statistical significance was evaluated by the *t*-test.

This study was approved by the Internal Review Board of Monmouth Medical Center (IRB # 17-011).

## 3. Results

During the 3-month post-intervention study period (November 2016 to January 2017), 179 of the 895 RBC transfusion orders (20%) triggered the alert. After excluding the duplicates, 144 alert orders (16% of the total) were included in our analysis. For 20 orders (13.88% of the 144), the alert prompted the ordering physician to cancel the transfusion. In the majority of cases (86.1%), the transfusion order was continued despite the alert. Of note, 24 of the active orders were not carried out (we did not investigate the rationale behind the decisions not to transfuse).

**Figure 2:**
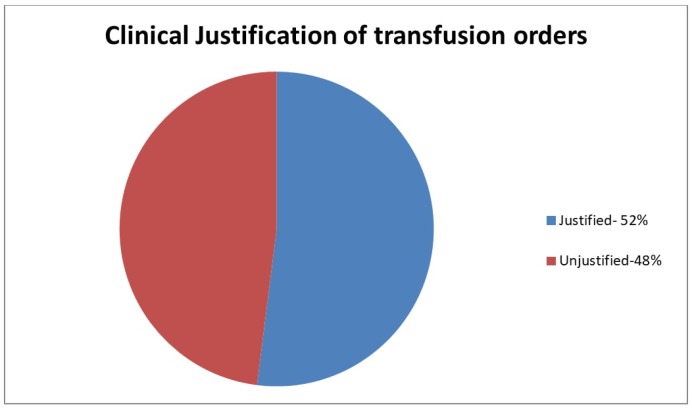
Chart review of the transfusion orders that were carried out despite the alert (justified versus unjustified).

**Figure 3:**
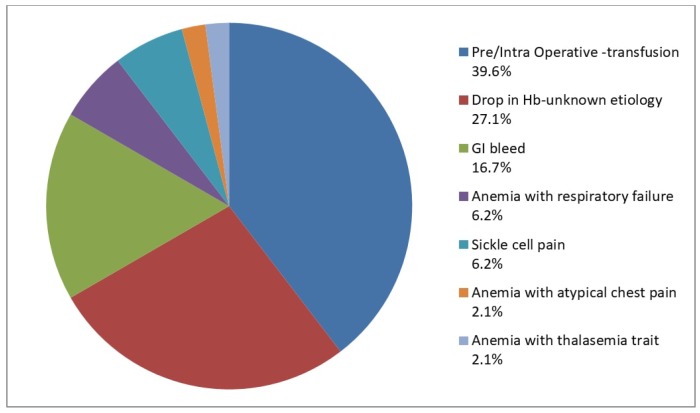
Clinical justifications selected from the drop-box or given under “other, please specify”) to deviate from the 7 g/dL transfusion threshold. Hb—hemoglobin; GI—gastrointestinal.

Next, we investigated the transfusions that were given despite the alert. Almost half of these transfusions (48%) could not be justified according to the AABB 2016 guidelines [8] (Figure 2). The most common causes of inappropriate RBC transfusions were (i) asymptomatic anemic patients who were either transfused pre-operatively or intra-operatively (29.2%); and (ii) patients whose hemoglobin dropped during hospitalization without an obvious cause (27.1%) (Figure 3). Other frequent causes included patients with gastrointestinal (16.7%) or intraoperative bleeding (10.4%) who were otherwise asymptomatic and hemodynamically stable, and whose hemoglobin remained above our transfusion threshold (7 g/dL for standard-risk patients).

**Figure 4:**
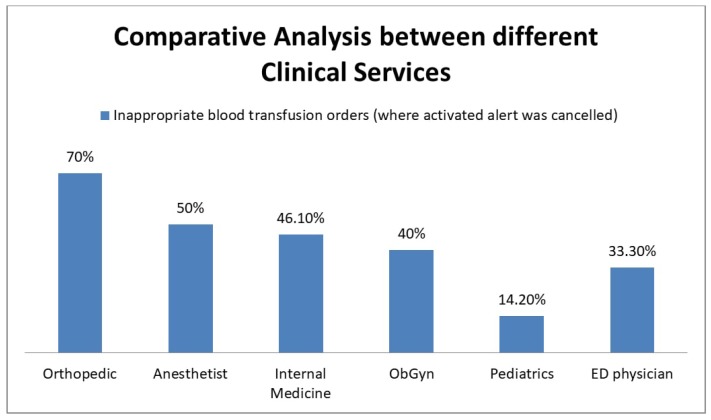
Inappropriate deviation from the 7 g/dL transfusion threshold according to the clinical service where the transfusion order was placed. ObGyn—Obstetrics and gynecology; ED—Emergency Department.

We also performed a comparative analysis of the effectiveness of the CDS between the different clinical services (Figure 4). The Orthopedics Department had the most inappropriate transfusions (70%), followed by Anesthesiology (50%) and Internal Medicine (46%). Nurse practitioners transfused inappropriately 55.3% (14/24) of the time, which was higher than physicians at 44.8% (34/76).

**Figure 5:**
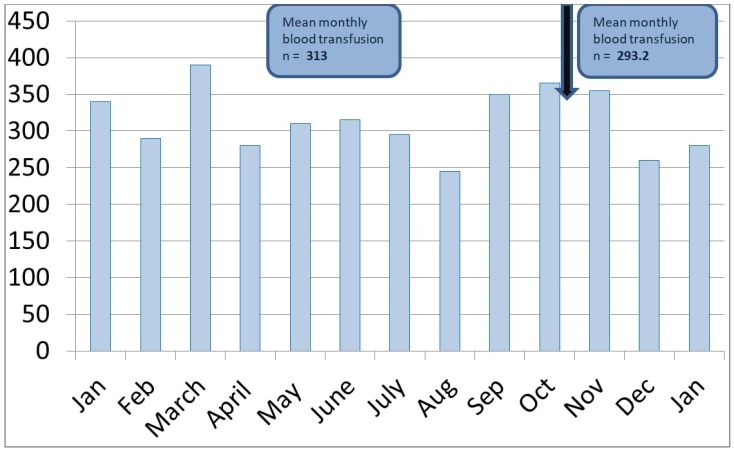
Monthly RBC transfusions before and after the implementation of the real-time clinical decision support (*y* axis denotes the units of packed red blood cells used between January 2016 and January 2017).

Last, we analyzed the monthly number of RBC transfusions in both the pre-intervention (January to October 2016) and post-intervention (November 2016 to January 2017) periods: after the implementation of the CDS, the average monthly blood utilization of RBC units (a 6.5% decrease) did not change significantly (Figure 5).

## 4. Discussion

The National Summit on Overuse (organized in 2012 by the Joint Commission and the American Medical Association’s Physician Consortium for Performance Improvement) named blood transfusion among the five most overused therapeutic interventions [9]. Indeed, most reports agree that 50% of the blood transfusions in the U.S. are unnecessary [2]. These transfusions not only do not benefit the patients but may also expose them to risks. In recognition of this problem, various strategies have been employed to improve blood utilization practices, including physician education based on retrospective utilization audits. An expert review of these efforts concluded that they do not modify future behavior without continuous reinforcement [6]. 

Monmouth Medical Center is a community-based teaching hospital. Beginning 2004, various steps have been taken to reduce the inappropriate use of blood products. In 2004, our hospital adopted a two-tier blood transfusion guideline, with a threshold of 8 g/dL for patients with symptomatic anemia [10]. In 2009, following the recommendations of the Society for the Advancement of Blood Management, the transfusion threshold was lowered from 8 g/dL to 7 g/dL in euvolemic, symptomatic chronic anemia patients, and physicians were advised to use single-unit transfusions [10,11]. To enforce these practices, nonconformant orders were placed on the approval list of the pathology resident on blood bank rotation. However, this practice was shortly abandoned because of the delays it caused [10]. Combined, these efforts reduced our average monthly RBC utilization from 410 in 2009 to 313 in 2016 (a 24% decrease) [12].

The introduction of EMRs paved the way for real-time CDS. An early version of this was implemented in 1987 at the Latter Day Saints Hospital, University of Utah, as a computerized ordering system with in-built guidance support called the HELP computer system [13]. This reduced the average transfusion hematocrit from 27.65 to 25.83 over a course of four years (1988–1992)—*p* value < 0.001 [13]. In 2010, The Stanford Hospital and Clinics implemented a real-time EMR-based CDS to guide transfusions [7]. This resulted in a 24% reduction in annual transfusions with a net savings of $1.6 million [6,7]. Similar results were reported by The University of California San Diego Health [14].

In November 2016 we incorporated a real-time transfusion support CDS (similar to that described by the Stanford group) into our EMR. During a 3-month post-intervention period, we saw a small (statistically not significant) decrease (6.5%) in our monthly RBC utilization; this should be compared with the more robust literature reports (19 to 24%) [7,14].

Admittedly, there are a number of limitations on our study. First, we collected alert transfusion orders for only three months post implementation of the CDS. Second, we relied on chart review for determining the appropriateness of transfusion which was not always clear.

Based on our experience, we conclude that real-time CDS built into the EMR per se may not be an effective way to improve blood transfusion practices at community-based hospitals. This system, however, may provide useful information for the Blood Utilization Committee to identify providers/clinical services that transfuse patients inappropriately despite the Best Practice Alert. Such providers/services can be targeted by a combination of education and monitoring.
